# *Perilla frutescens* L.: a dynamic food crop worthy of future challenges

**DOI:** 10.3389/fnut.2023.1130927

**Published:** 2023-06-01

**Authors:** Chubasenla Aochen, Amit Kumar, Sandeep Jaiswal, Kekungu-u Puro, Philanim Wungmarong Shimray, Subarna Hajong, Rumki Heloise Ch Sangma, Sentibenla Aochen, Banshanlang Iangrai, Bijoya Bhattacharjee, Lemnaro Jamir, Thejangulie Angami, Arunava Pattanayak, Vinay Kumar Mishra

**Affiliations:** ^1^The ICAR Research Complex for North Eastern Hill Region (ICAR RC NEH), Umiam, India; ^2^National Bureau of Plant Genetic Resources, Indian Council of Agricultural Research (ICAR), New Delhi, India; ^3^Fazl Ali College, Mokokchung, Nagaland, India; ^4^Indian Council of Agricultural Research (ICAR) - Indian Institute of Agricultural Biotechnology, Ranchi, India

**Keywords:** fatty acid biosynthesis, climate resilience, Perilla, nutrition, bioavailability

## Introduction

Perilla (*Perilla frutescens* L.) is an annual aromatic plant of the Lamiaceae family cultivated and widely consumed in most Asian countries. The cultivated tetraploid species *P. frutescens* L. Britton include var. *frutescens*, a regular vegetable crop and an oil crop, and var. *crispa*, known for both medicinal and nutritional value. It is commonly known as beefsteak plant, perilla mint, Chinese basil, or purple mint. Leaves are used as culinary herb, seedlings and seed oils are added to salads while seeds are used as garnish or condiment. Perilla is also integral to Chinese traditional medicine with proven cures and treatments (1).

## Nutrition and bioactives content

Perilla seed oil is considered one of the best plant sources of omega fatty acids, with about 53–62% α-linolenic acid (ALA, 18:3, ω-3), 10–13% linoleic acid (LA, 18:2, ω-6) and 11–16% oleic acid (18:1, ω-9) and mean ω-6/ω-3 ratio of about 0.20 (2, 3). Moreover, it hosts adequate concentrations of minerals, vitamins, amino acids, flavonoids and polyphenols in different edible forms of seeds and leaves, making it a versatile nutraceutical crop (Table 1). Seeds contain significantly higher essential amino acids and mineral content compared to leaves. Seeds also provide higher Fe, Mg, Cr and comparable amounts of protein and P among commercial oilseeds, *viz*. mustard, linseed, groundnut, and sunflower (4). Fresh leaves have higher β-carotene and lutein content as compared to common carotenoids-rich vegetables like carrots, spinach, broccoli, lettuce, parsley and pumpkin leaves (5, 6). Drying perilla leaves effectively boosted the concentrations of flavonoids without compromising on flavor, and thereby emphasize the efficiency of bioactives in dried forms (1, 3, 7).

**Table 1:** Nutritional composition of Perilla in edible forms of leaves and seeds (per 100 g) (3).

**Nutrient**	**Leaves**			**Seed**	
	**Raw**	**Blanched**	**Steamed**	**Dry**	**Roasted**
Moisture (g)	84.3	89.6	87.3	4.6	0.9
Protein (g)	4.46	3.93	4.37	22.7	23.6
Fat (g)	0.5	0.6	0.55	39.7	36.2
Ash (g)	1.85	1.07	1.71	3.72	3.78
Carbohydrate (g)	8.89	4.8	6.07	29.3	35.5
Fiber (g)	5.69	3.9	4.7	22	22
Energy (Kcal)	47	32	37	530	530
Ca (mg)	296	226	265	391	416
Mg (mg)	151	56	111	254	315
Fe (mg)	1.91	1.39	2.42	7.74	7.5
Cu (mg)	0.159	0.132	0.164	1.21	1.86
Mn (mg)	0.371	2.253	1.68	3.69	4.61
Zn (mg)	0.44	1.3	1	4.8	6.78
P (mg)	83	72	77	716	726
K (mg)	421	196	411	583	599
Se (μg)	2.52	2.07	2.26	1.16	6.1
Mo (μg)	11.5	11.4	11.8	14.02	82.7
Iodine (μg)	1.76	0.51	0.7	-	2.57
Isoleucine (mg)	157	149	133	769	770
Leucine (mg)	339	300	277	1,549	1,496
Lysine (mg)	227	216	206	938	864
Methionine (mg)	67	52	51	458	449
Phenylalanine (mg)	221	195	184	1,157	1,138
Threonine (mg)	190	167	157	798	849
Tryptophan (mg)	50	44	45	225	120
Valine (mg)	210	190	180	1,023	1,137
Histidine (mg)	78	74	69	625	623
Vitamin A (μg)	630	587	601	3	2
β-Carotene (μg)	7,565	7,040	7,207	33	29
Thiamin (B1) (mg)	0.329	0.045	0.138	0.353	0.528
Riboflavin (B2) (mg)	0.508	0.161	0.421	0.759	0.311
Niacin (B3) (mg)	0.3	0.064	0.174	2.58	4.363
Pantothenic acid (B5) (mg)	0.255	0.063	0.255	0	0.087
Biotin (B7) (μg)	-	-	-	1.65	1.65
Folate (B9) (μg)	150	38	64	101	63
Ascorbic acid (C) (mg)	2.73	0.13	1.05	-	-
α-tocopherol (mg)	1.07	1.45	2.02	1.4	1.15
β-tocopherol (mg)	0.03	0.01	0.03	-	-
γ-tocopherol (mg)	0.03	-	-	25.37	8.36
δ-tocopherol (mg)	-	-	-	0.44	0.27
Phylloquinone (μg)	786.6	655.88	773.81	-	-
Apigenin (mg)	190.1 (dried)	-	-	3.97	-
Luteolin (mg)	158.1 (dried)	-	-	9.96	-
Rosmarinic acid (mg)	3,545 (dried)	-	-	368	335.6
Caffeic acid (mg)	8.1 (dried)	-	-	5.96	1.93

## Optimal factors

Perilla thrives in higher altitudes of semi-tropical environments with relatively lower mean annual temperature with suitable humidity. Transplanting seedlings during cooler spring season produced taller plants, higher leaf area index, and greater fresh weight of aerial parts, and harvesting of leaves 110 to 120 days after transplanting is considered optimal time to derive highest quantity of leaf perillaldehyde (8). Harvesting of vegetative crop at the end of the second month after sowing is recommended for an optimal compromise between yield and nutritional value (9).

## Value-added prospects

Perillaldehyde, the major essential oil of perilla, contributes to aroma and exudes its distinctive flavor in baked foods, pickled vegetables, sauces, salads, meats, puddings and beverages (10). Perillaldehyde has also been judged safe by FAO/ WHO Joint Expert Committee on Food Additives (JECFA) (11). Perilla-fortified products include meatballs from perilla seed meals as fat replacements, traditional pepper oil sauce with 10% replaced perilla oil, yogurt with 10% substituted perilla oil, muffins with freeze-dried perilla leaves, chewable tablets with 8% powdered blanched leaf with improved nutrition profiles (12–16). Premium quality perilla tea is prepared with roasted perilla leaves (180^o^C for 20 min), with optimal extraction of polyphenols and flavonoids and antioxidant functions (17). Perilla plant flowers when other blossoming plants are scarce, and this aspect of phenology is touted to provide alternative resources in achieving higher honey production with an autumn harvest. Moreover, this extends the egg-laying period of queen bee which thereby, lessen the extent of losses from the parasite *Varroa destructor* (Anderson & Trueman) (18). Intercropping of tea plants with aromatic plants perilla and basil is reported to improve tree crown formation and vigor of young leaves, and enhance catechins content, thereby improving the yield and quality of tea (19).

## Bioavailability of dietary ω-fatty acids

ALA is an essential fatty acid due to the deficiency of human ω-3 fatty acid desaturase enzyme. The metabolic products of LA and ALA include long chains ω-6 PUFA arachidonic acid (C20:4) and ω-3 acids eicosapentaenoic acid (EPA, C20:5) and docosahexaenoic acid (DHA, C22:6), found abundantly in fish oil, respectively. The rate of synthesis of AA and EPA is directly dependent on the availability of substrate LA and ALA since the same enzymes catalyze the conversion reactions (20). Dietary ALA also undergoes mitochondrial β-oxidation, therefore, its use as a substrate for conversions to EPA and DHA becomes more limited. The consumption of healthy PUFA is paradoxical. The increased dietary consumption of LA-rich oils of soybean, safflower, sunflower and corn in the West accounts for up to 15 times higher intake of LA than ALA, shooting up the ω-6/ω-3 ratio to 16–20:1 against a dietary recommendation of ≤ 4:1, and has in turn increased disease risks (21). The intake of ALA-rich oils like perilla oil, therefore, becomes significant.

## Fatty acid biosynthesis and abiotic stress tolerance

Two important transcription factor binding sites, AP2 and B3, responsible for β-ketoacyl-acyl carrier protein synthase II (*kasII*) and fatty acid desaturase-3 (*fad3*) genes, respectively, in perilla seeds was highlighted; increase in these genes during oil accumulation phase is directly associated with ALA biosynthesis. Moreover, AP2 and B3 transcription factor families are also integral to abiotic stress such as drought and low temperature environments, where increase of *kasII* and *fad3* genes was seen (22). One significant drought tolerance response is to incorporate newly synthesized PUFA into membrane lipids through gene action (23). This holds relevance for perilla crop which is a short-day plant, requiring longer nights for induction of flowering and low temperatures for PUFA production (24). Perilla crop, which is accustomed to mountainous or terrains of higher altitude is, therefore, seen to thrive in lower temperature and drier conditions during seed maturation. This linked duality of functions of perilla for both fatty acid production and abiotic stress tolerance gives impetus to its importance in both nutrition and abiotic stress response.

## Advances in nutritional research

PrLeg, an 11S legumin-like storage protein isolated from developing seeds of perilla, and containing high levels of sulfur-containing amino acids when transformed into potato plant could produce transgenic potato lines that could accumulate high amount of PrLeg transcript with 3-fold increase in methionine content (25). Transgenic perilla lines was developed with overexpression of γ-tocopherol methyltransferase (γ-TMT) gene leading to efficient conversion of γ-tocopherol to α-tocopherol and dramatic increase in seed α-tocopherol (26). Using metabolomics studies, glycolic acid has been highlighted as potential biomarker for perilla seeds to distinguish between perilla from different geographical regions. While this pioneering study is restricted to Korea and Japan, the potential to trace and secure the regional authenticity of perilla sources to ensure global trade safety is promising (27). SSR markers associated with total fatty acid content, ALA and oleic acid have been deciphered (28). Development of such markers linked to target genes can be utilized in selecting superior germplasms and breeding for superior oil quality through marker-assisted selection. Besides, 43 genes involved in fatty acid and triacylglycerol synthesis in perilla seed oil was elucidated recently (29). These studies provide critical information not only for studies on mechanisms involved in ALA synthesis but also for biotechnological production of ALA in other oilseeds.

## Advances in health research

In very active female athletes, daily intake of perilla oil as ω-3 fatty acid supplement improved their gut microbiota diversity including butyrate-producing bacteria and subdued the growth of *Proteobacteria*, besides supplying additional energy (30). Methanolic extracts of seed, leaves and stalk could exhibit antiproliferative activities against human non-small cell lung A549 cancer cells (31). Perilla anthocyanins could induce apoptosis in human cervix Adenocarcinoma Hela cells at 300 μg/mL (32). The addition of perilla oil to drug treatments (epirubicin combined with paclitaxel) in breast cancer patients could effectively improve the quality of life and reduce adverse reactions rates (33). Unlike PUFA-rich safflower and fish oils, perilla oil is reported to regulate brown and white adipose tissue metabolism and prevent the accumulation of body fat and regulation of glucose metabolism (34). Luteolin-rich perilla seed meal fractions (25–100 μg/mL) potentially induce anti-inflammatory action against spike glycoprotein S1 of SARS-CoV-2-induced inflammation in A549 lung cells during incidence of long-COVID by downregulating JAK1/STAT3-inflammasome-dependent inflammatory pathway (35). Perilla pomace extract in cosmetic formulations could exhibit collagenase inhibitory effects at 400 μg/mL and anti-melanogenic effect on B16F10 melanoma cells without inducing cell cytotoxicity, and with clinical improvements in skin elasticity and reduced hyperpigmentation (36).

## Looking ahead

The potential of Perilla as a future food crop is immense. In non-Asian countries, studies on perilla or its dietary and culinary use is sporadic. Perilla was identified as new genus in Turkey in 2002, and in Bosnia and Herzegovina only in 2018 (37, 38). Reportedly perilla is considered an invasive plant in natural areas across the mid-Atlantic region of United States (39). The nutritional potential and the versatility of perilla crop is still to be realized in non-Asian countries.

Major perilla growing countries in Asia have standardized cultivation practices suited to their climatic conditions. In India, cultivation is generally at household level with no established cultivation practices. Released varieties are scanty in the absence of effective selection criteria. Several genotypes of Perilla still occur as cultivars in farmers' fields in several East Asian regions (40, 41). Integrating sporadic research data on perilla across different climatic zones and cultivation practices can help address certain challenges. Moreover, in many tribal pockets in India, perilla is restricted only to traditional culinary use; technologies are lacking to upscale perilla resources on a commercial scale.

Perilla is generally a long duration crop, and colder temperatures during flowering time reportedly affect seed setting, along with seed shattering around crop maturation (41). Seed shattering is an evolutionary trait quite common to weedy species. At the same time, the difficulty in distinguishing between Perilla cultivars and weedy species is a challenge. While morphological distinctions are described for cultivated varieties and their weedy forms, obscurity is still observed in large populations (42).

Core collections are lacking in Perilla. The first core collection with 44 accessions based on SSR markers and morphological characteristics is recently reported from South Korea, accounting for 11% of the Perilla collection (43). Unlike traditional crops, in a versatile crop like perilla, the nutritional quality and metabolic content become the most important phenotypic traits along with agromorphological characteristics for development of core collection, and molecular data ensures reliable genetic variations between germplasm. Development of integrated core collections, therefore, is essential for future breeding programs.

Genes coding various transcription factors like WRINKLED, FUSCA3, LEAFY COTYLEDON1, ABSCISIC ACID INSENSITIVE3, along with various enzymes for PUFA biosynthesis and acyl-related enzymes is identified in Perilla (44). Besides their functional validation, the identification of epigenetic control of FAs biosynthesis is also important to understand the vast network of FA biosynthesis in perilla. The complete chloroplast genome of *P. frutescens* (L.) Britton var. *frutescens*, 153 Kb in length, has been assembled with 127 annotated genes (45). A near-complete chromosome-level assembly (99.2%) of *P. frutescens* cultivar Hoko-3 (Pfru_yukari_1.0) is established with a genome size of 1.258 Gb and 72,983 functionally annotated genes (46). Advances in perilla genomics are unique resources for future genome editing studies, and metabolic engineering of perilla to enhance the biosynthesis of important metabolites.

While the therapeutic uses of perilla is documented, ample preclinical studies *in vitro* and *in vivo* and in animal models provide potential biological evidence for preventive therapy. More investigations on the therapeutic findings in clinical settings are required for validation of their efficacy for efficient product development and ethical use.

This ancient underutilized food crop requires contemporary interventions. Variations in the performance of perilla crop in terms of geographical influences, sowing time, sampling time has been established. New leads in the adaptation and performance of perilla under natural stress conditions like light fluctuations, salinity, acidic soils, waterlogging, high temperatures etc. are still lacking for climate-smart breeding programmes. New emerging pests and diseases also pose new threats. A new fungal disease of Perilla, stem blight caused by *Corynespora cassiicola*, reported from Korea affected greenhouse production (47). The shifts and extremities in the climate pattern can affect the phenology of crop and thereby crop and vegetative yield. Intercropping with phenolics-rich perilla crop requires attention given its potential to decrease disease index and incidence rate (48). The existence of various abiotic and biotic stressors in perilla crop calls for concurrent research and management strategies across different geographical and climatic factors. The potential of Perilla as a future food crop will be realized when the vagaries of a changing climate on the crop are also addressed.

## Author contributions

CA planned, contributed, and revised the manuscript. K-uP contributed and provided technical guidance. AK, SA, SH, RS, LJ, TA, BB, and BI contributed to different subheadings. SJ and PS contributed and compiled the manuscript. AP and VM provided objective insights. All authors contributed to the article and approved the submitted version.
